# Jacalin Has Chemopreventive Effects on Colon Cancer Development

**DOI:** 10.1155/2017/4614357

**Published:** 2017-06-06

**Authors:** Thais Herrero Geraldino, Patricia Modiano, Luciana Chain Veronez, Milena Flória-Santos, Sergio Britto Garcia, Gabriela Pereira-da-Silva

**Affiliations:** ^1^Postgraduate Program in Basic and Applied Immunology, Ribeirão Preto School of Medicine, University of São Paulo, Ribeirão Preto, SP, Brazil; ^2^Ribeirão Preto College of Nursing, University of São Paulo, Ribeirão Preto, SP, Brazil; ^3^Postgraduate Program in Public Health Nursing, Ribeirão Preto College of Nursing, University of São Paulo, Ribeirão Preto, SP, Brazil; ^4^Department of Pathology, Ribeirão Preto School of Medicine, University of São Paulo, Ribeirão Preto, SP, Brazil

## Abstract

Colorectal cancer, which is one of the most common causes of cancer-related deaths worldwide, has a slow natural history that provides a great opportunity for prevention strategies. Plant-derived natural products have received considerable attention because of their inherent colorectal cancer chemopreventive effects. The plant lectin jacalin specifically recognizes the tumor-associated Thomsen-Friedenreich antigen and has antiproliferative effects on human colon cancer cells, highlighting its potential antitumor activity. To evaluate jacalin's potential application in colorectal cancer chemoprevention, we studied its effects on the early stages of carcinogenesis. Balb/c mice were given 4 intrarectal deposits of 0.1 ml solution of Methyl-N′-Nitro-N-Nitroso-Guanidine (5 mg/ml) twice a week (with a 3-day interval) for 2 weeks. Starting 2 weeks before carcinogen administration, animals were treated orally with jacalin (0.5 and 25 *μ*g) three times a week (on alternate weekdays) for 10 weeks. We show that jacalin treatment reduced the number of preneoplastic lesions in carcinogen-exposed mice. This anticarcinogenic activity was associated with decreased colonic epithelial cell proliferation and stromal COX-2 expression and with increased intestinal production of TNF-*α*. Our results demonstrate that jacalin is able to modulate the early stages of colon carcinogenesis and emphasize its promising chemopreventive activity in colorectal cancer.

## 1. Introduction

Colorectal cancer (CRC) is one of the most common causes of cancer-related deaths worldwide [1]. CRC development is a slow process that has a natural history of transition from normal crypts through adenoma to overt adenocarcinoma, providing a great opportunity for prevention and intervention strategies. Although the early detection of invasive lesions and precursor adenomatous polyps reduces morbimortality, only a few cases of CRC have been diagnosed in early stages [2]. Currently, most attention has focused on screening for chemopreventive agents to reduce the number of CRC patients.

Cancer chemoprevention uses natural, synthetic, or biological substances to reverse, suppress, or prevent the initial phase of carcinogenesis or the progression of neoplastic cells to cancer [3]. Epidemiological studies have suggested that dietary nutrients from fruits and vegetables contribute to keeping balanced cell proliferation and preventing carcinogenesis. Moreover, phytonutrients have received considerable attention because of their low cost and wide safety. Experimental evidence has established the potential colorectal cancer chemopreventive effects of plant-derived natural products [4, 5].

Plant lectins, carbohydrate-binding proteins distributed in a variety of plant species, are gaining clinical implications because of their potential antitumor properties. These molecules can recognize the altered glycosylation of cancer cells, mostly inhibiting proliferation and inducing tumor cell death [6–8]. Jacalin, a noncytotoxic lectin extracted from jackfruit* (Artocarpus heterophyllus) *seeds, specifically recognizes the Thomsen-Friedenreich antigen (TF-Gal*β*1-3GalNAc), which is expressed in more than 85% of human carcinomas, and has antiproliferative effects on human colon cancer cells, highlighting its potential antitumor activity [9]. Over the last few years, jacalin has emerged as a candidate carrier for delivery of cancer therapeutics [10, 11]. However, to date, its in vivo direct antitumor activity has not been investigated. To evaluate jacalin's potential application in colorectal cancer chemoprevention, we studied its effects on the early stages of carcinogenesis.

## 2. Materials and Methods

### 2.1. Jacalin Purification

Jacalin was isolated from the seeds of* Artocarpus heterophyllus* by affinity chromatography on immobilized D-galactose Sepharose column, as previously described [12]. Sample purity was evaluated by 12% SDS/PAGE under reducing conditions and was based on the presence of protein bands of 11.4 and 14.7 kDa and the absence of 13 kDa band corresponding to Artin-M. Protein concentration was measured by BCA protein assay kit (Pierce). Jacalin diluted in PBS was used in the assays.

### 2.2. Mice and Treatment Protocol

**Figure 1:**
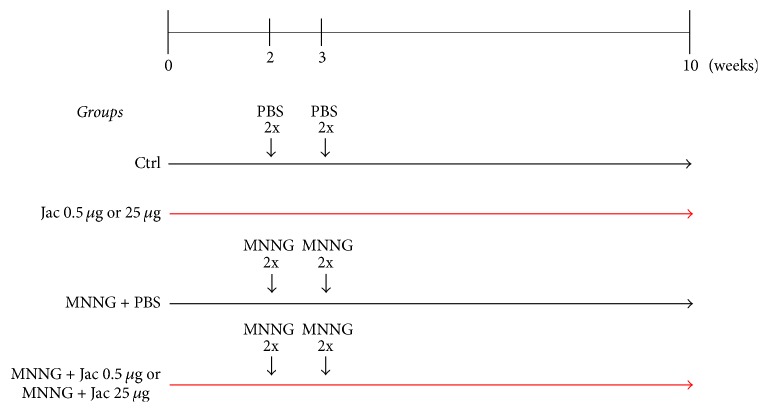
Treatment protocol. Mice received 4 successive intrarectal deposits of 0.1 ml of PBS or MNNG solution (5 mg/ml) twice a week for 2 weeks. Starting 2 weeks before carcinogen administration, animals were treated orally (0.1 ml/mouse) with PBS (→) or jacalin (0.5 and 25 *μ*g) (→) three times a week for 10 weeks. After 10 weeks of treatment, mice were euthanized, and the colons were removed, fixed, and processed for histological and immunohistochemical analysis.

6-Week-old Male Balb/c mice were housed at the Animal Facility of Ribeirão Preto College of Nursing, the University of São Paulo (Ribeirão Preto, SP, Brazil). Animals were maintained at 22 ± 2°C with 55% humidity on a 12 h–12 h light/dark cycle, receiving chow and water ad libitum. The experimental protocol was performed in accordance with the guidelines of the Animal Care and Ethics Committee of the University of São Paulo. After 2 weeks of acclimation period, mice were randomly distributed into six groups (8 mice each) and were injected with PBS or Methyl-N′-Nitro-N-Nitroso-Guanidine (MNNG, Sigma-Aldrich, Louis, MO, USA) (4 successive doses of MNNG 5 mg/ml; intrarectal deposits of 0.1 ml) twice a week (separated by a 3-day interval) for 2 weeks, as illustrated in Figure 1. Jacalin treatment started 2 weeks before carcinogen administration. Mice were treated orally (0.1 ml/mouse) with jacalin (0.5 and 25 *μ*g) three times a week (on alternate weekdays) for 10 weeks (Figure 1). At the end of the 10th week, mice were euthanized by CO_2_ exposure, and the colons were removed, longitudinally opened, fixed flat in 4% neutral paraformaldehyde buffer, and processed for histological and immunohistochemical analysis. Small pieces (5 mm) of the distal colon were removed for cytokine analysis.

### 2.3. Histological Analysis

Colon tissue samples were sectioned, stained with H&E, and analyzed under light microscopy in a double-blind manner by two histopathologists. Dysplastic aberrant crypt foci (ACF) were identified at 200x magnification and counted at 400x magnification, according to previously reported criteria [13, 14]. In brief, dysplastic lesions were assessed in transversal sections, and dysplastic patterns ranging from mild to moderate dysplasia were counted in 20 microscopic fields for each carcinogen-treated animal. The dysplasia-i (index) was expressed as the ratio between dysplastic and nondysplastic crypts. Relative values for ACF-i (index) were calculated as their total number per mm^2^.

### 2.4. Immunohistochemical Procedures

Primary antibodies were obtained from Novocastra Laboratories (Newcastle, UK). The streptavidin-biotin method (Novostain Universal Detection Kit, Novocastra Laboratories, Newcastle, UK) was used with anti-PCNA (clone PC10 at 1 : 100), anti-caspase-3 (clone JHM62 at 1 : 50), and anti-COX-2 (clone 4H12 at 1 : 100) monoclonal antibodies. Paraffin-embedded colon tissue sections (4 *μ*m) were processed as described previously [15] and counterstained with hematoxylin. A tonsil slide was used for control.

### 2.5. PCNA, Cleaved Caspase-3, and COX-2 Analysis

To estimate colonic cell proliferation or death and colonic inflammation, the colon tissue slices were immunostained with anti-PCNA, anti-Caspase-3, or anti-COX-2 antibodies, respectively. At least 20 perpendicular well-oriented normal-appearing colon crypts per animal were examined under light microscopy in a double-blind manner by two investigators. The index of colonic crypt cells expressing proliferating cell nuclear antigen (PCNA-i) was calculated from the ratio of positively stained nuclei to a total number of nuclei counted, whereas caspase-3 and COX-2 indexes (Caspase-3-i and COX-2-i) were expressed as the number of stained cells in the intercrypt spaces.

### 2.6. Cytokine Quantification

For cytokine detection, colon was homogenized in 1 ml ice-cold homogenization buffer (PBS with protease inhibitor cocktail, Roche). The supernatant was collected by centrifugation (15 minutes, 5.000 rpm, 4°C) and stored at −20°C until use. The levels of IL-1*β*, IL-6, IL-10, IL-12p70, TNF-*α*, IFN-*γ*, and TGF-*β* were measured by ELISA (BD OptEIA™), according to the manufacturer's protocol. Cytokine concentration was determined with reference to a standard curve of murine recombinant cytokines and normalized to the weight of the colons.

### 2.7. Statistical Analysis

Data were analyzed using the statistical program GraphPad Prism 5.0. For all experiments, one-way ANOVA (Kruskal–Wallis) and Dunn's multiple comparison post hoc tests were applied for samples with nonnormal distribution, and one-way analysis of variance was used for those with normal distribution. A probability of *P* < 0.05 was considered significant. Results were expressed as mean ± SEM.

## 3. Results

### 3.1. Aberrant Crypt Foci Quantification

**Figure 2:**
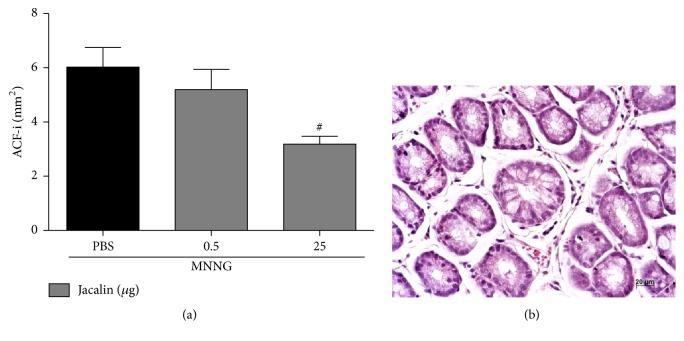
*Jacalin treatment reduces the number of aberrant crypt foci in mice colon*. (a) Quantification of aberrant crypt foci. The aberrant crypt foci index (ACF-i) shows the number of lesions per mm^2^. (b) Representative histological image of a characteristic ACF with compressed cryptal luminal opening (400x magnification, scale bars represent 20 *μ*m). Statistical analysis: one-way analysis of variance. ^#^Compared to PBS group. ^#^*P* < 0.05.

To evaluate whether jacalin has a chemopreventive activity in carcinogen-exposed mice, colonic preneoplastic lesions—aberrant crypt foci (ACF)—were counted 10 weeks after the first injection of MNNG. We observed that all MNNG-exposed animals developed ACF in the colon (Figure 2(a)). However, the number of ACF was significantly lower in the group treated with the higher dose of jacalin (25 *μ*g) when compared to the untreated group. No difference was observed between untreated group and that treated with the lower dose of jacalin (0.5 *μ*g). These results demonstrate a potential chemopreventive activity of jacalin against colon cancer development.

### 3.2. Jacalin Effects on Cell Proliferation and Apoptosis

**Figure 3:**
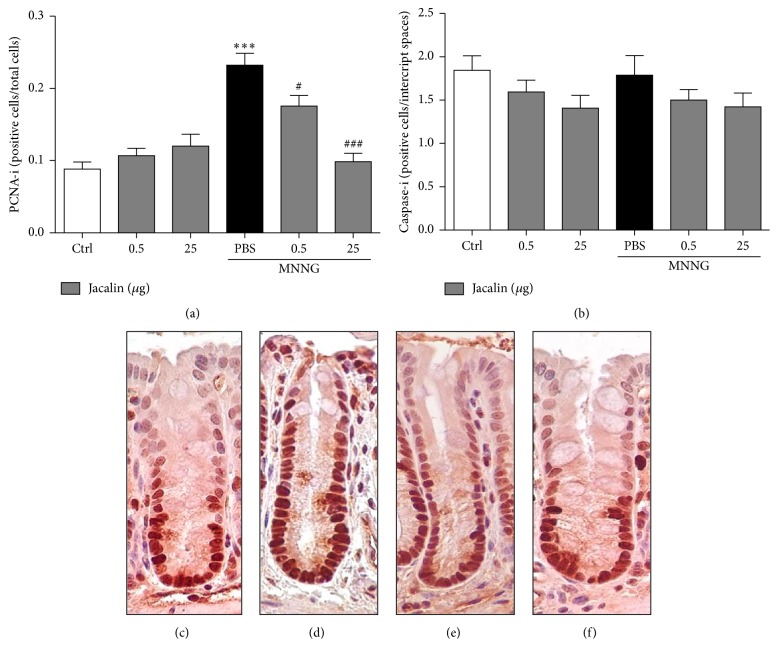
*Jacalin treatment affects colonic epithelial cell proliferation but not apoptosis*. (a) Proliferative activity in the colon was assessed by using proliferating cell nuclear antigen (PCNA) antibody. The index of colonic crypt cells expressing proliferating cell nuclear antigen (PCNA-i) was calculated from the ratio of positively stained nuclei to a total number of nuclei counted. (b) Apoptotic cells were detected by caspase-3 immunoreactivity in colonic stromal cells. Apoptosis index shows the number of stained cells in the intercrypt spaces. (c) Representative immunohistochemistry images of proliferating cells of the control group; (d) MNNG-exposed group; (e) MNNG-exposed group treated with jacalin 0.5 *μ*g; and (f) MNNG-exposed group treated with jacalin 25 *μ*g. Results are expressed as mean ± SEM for each group. Statistical analysis: (a) one-way analysis of variance and (b) one-way ANOVA (Kruskal-Wallis) and Dunn's multiple comparison post hoc tests. ^*∗*^Compared to control group. ^#^Compared to PBS group. ^#^*P* < 0.05; ^*∗∗∗*^  or ^###^*P* < 0.001.

Given that increased cell proliferation and apoptosis have been associated with development of colon tumors [16], we next evaluated whether jacalin interferes with colonic epithelial cell proliferation and apoptosis rates in the “normal-appearing” crypts of carcinogen-exposed mice. The MNNG-exposed group showed a significant increase of colonic mucosal cell proliferation as demonstrated by PCNA immunoreactivity (PCNA-positive epithelial cells, Figure 3(a)). Treatment with jacalin reduced PCNA index; the group treated with the higher dose showed lower PCNA index compared to that treated with the lower dose. On the other hand, the apoptosis indexes (cleaved caspase-3-positive epithelial cells) were similar in all groups (Figure 3(b)). These results show that jacalin has antiproliferative activities on colonic epithelial cells.

### 3.3. COX-2 Expression and Cytokine Production

**Figure 4:**
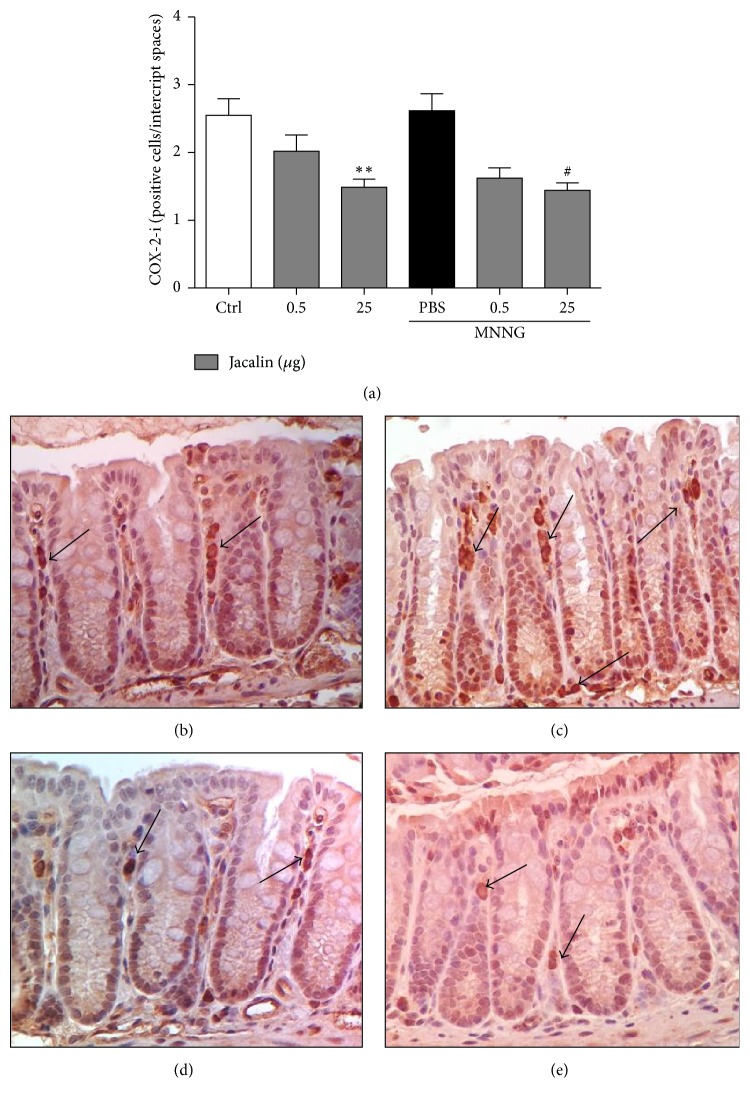
*Downregulation of COX-2 expression in colonic tissue of jacalin-treated mice*. (a) Cyclooxygenase-2 expression was evaluated using anti-COX-2 antibody. Morphometric analysis showed decreased COX-2 expression in both MNNG-exposed and unexposed animals treated with the higher dose of jacalin. (b) Representative immunohistochemistry images of COX-2 positive cells in the intercrypt spaces of the control group; (c) MNNG-exposed group; (d) MNNG-unexposed group treated with jacalin 25 *μ*g; and (e) MNNG-exposed group treated with jacalin 25 *μ*g. COX-2 index was calculated as the number of stained cells in the intercrypt spaces and is expressed as mean ± SEM for each group. Statistical analysis: one-way ANOVA (Kruskal-Wallis) and Dunn's multiple comparison post hoc tests. ^*∗*^Compared to control group. ^#^Compared to PBS group. ^#^*P* < 0.05; ^*∗∗*^*P* < 0.01. Arrows show COX-2-positive cells.

Cyclooxygenase-2 and proinflammatory cytokines are important cofactors in the pathogenesis of cancer [17]. These observations prompted us to investigate COX-2 expression and cytokine production in the colonic tissue of jacalin-treated mice. Administration of the higher dose of jacalin decreased the number of COX-2 positive cells in both MNNG-exposed and control (MNNG-unexposed) groups (Figure 4).

**Figure 5:**
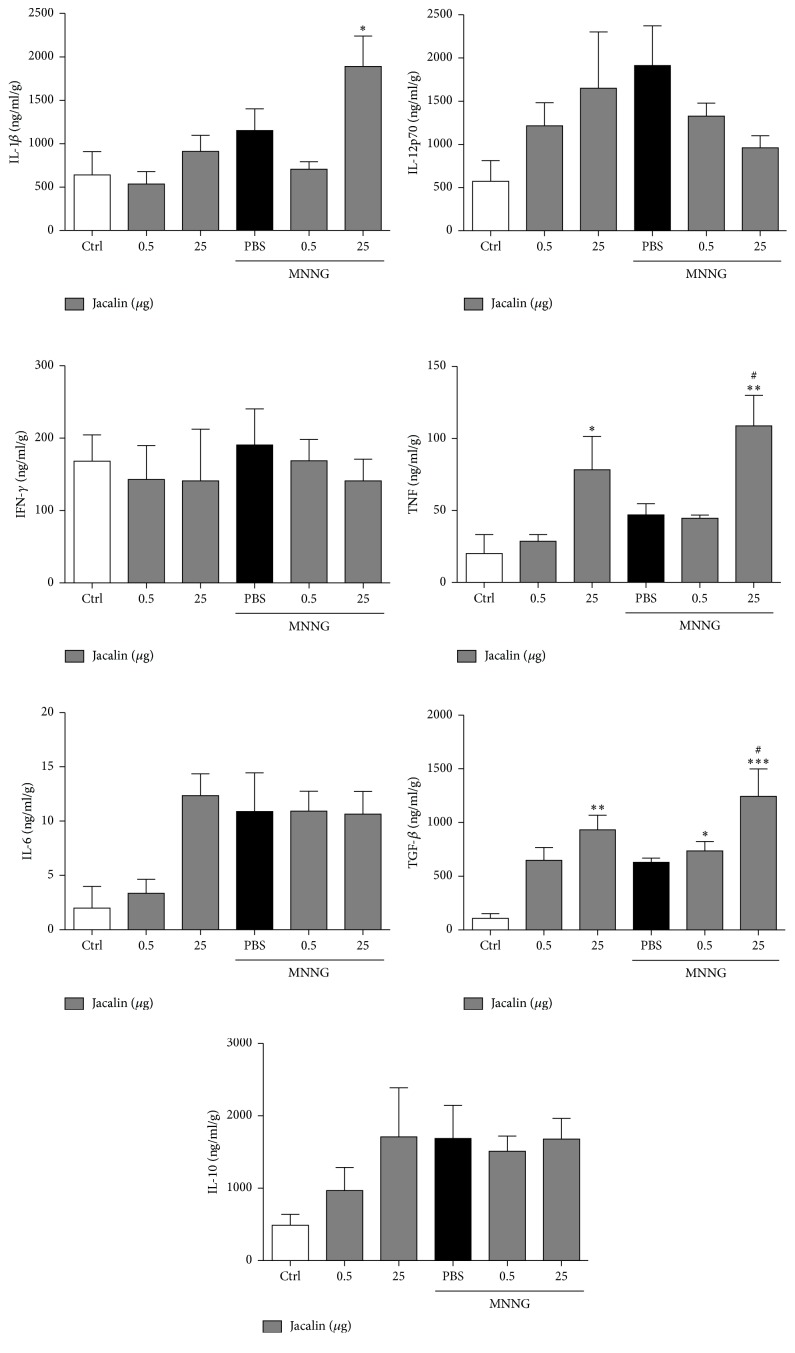
*Jacalin treatment affects intestinal cytokine production*. MNNG-exposed mice were treated with jacalin (0.5 and 25 *μ*g) and proinflammatory and anti-inflammatory cytokine levels in colon homogenates were determined by ELISA. Jacalin treatment led to a significant increase in TNF and TGF-*β*1 levels in colonic tissue. Results are expressed as mean ± SEM for each group. Statistical analysis: one-way analysis of variance. ^*∗*^Compared to control group; ^#^compared to PBS group. ^*∗*^  or ^#^*P* < 0.05; ^*∗∗*^*P* < 0.01 and ^*∗∗∗*^*P* < 0.001.

To study the effects of jacalin on the production of anti- and proinflammatory cytokines, TNF-*α*, IFN-*γ*, IL-1*β*, IL-12p70, IL-6, TGF-*β*, and IL-10 contents in colon homogenates from carcinogen-exposed mice were determined by ELISA. Compared to the control group, MNNG administration did not alter the cytokine profile. In contrast, jacalin administration (25 *μ*g) to both MNNG-exposed and unexposed mice led to increased levels of the pro- and anti-inflammatory cytokines TNF-*α* and TGF-*β*, respectively (Figure 5). No differences in the production of IFN-*γ*, IL-1*β*, IL-12p70, IL-6, and IL-10 were observed among the groups. These results indicate that jacalin modulates COX-2 expression, TNF-*α*, and TGF-*β* production in colonic tissue during carcinogenesis.

## 4. Discussion

In the present study, using a mouse model of colon carcinogenesis induced by Methyl-N′-Nitro-N-Nitroso-Guanidine (MNNG), we evaluated the effects of jacalin on the early stages of colonic tumorigenesis. Our results demonstrated that the lectin has chemopreventive activity, as shown by the reduced number of preneoplastic lesions (ACF) in carcinogen-exposed mice upon lectin treatment. To our knowledge, this is the first study to show that jacalin interferes with development of preneoplastic lesions in vivo.

Alterations in cell proliferation and apoptosis balance may lead to an increased risk of developing cancer [18]. Previous studies have already demonstrated that jacalin has an antiproliferative activity on human carcinoma cell line [9]. In agreement with these findings, we show that jacalin administration to carcinogen-exposed mice decreased colonic epithelial cell proliferation. These observations emphasize that lectins that specifically bind to the TF antigen, which is highly expressed in malignant and premalignant epithelia [19, 20], can affect intestinal epithelial cell proliferation [21] and thus have important functional effects in the colonic epithelium.

Cyclooxygenases (COX) are important regulatory enzymes in tumorigenesis. Overexpression of COX-2 and its products have been associated with various premalignant and malignant lesions of the gastrointestinal tract [22–24], and COX-2 has been posited as a potential biomarker for cancer risk and poor prognosis [24–27]. Indeed, patients bearing tumors with high levels of COX-2 have shown significantly reduced survival [28, 29], while a 40–50% reduction of colon cancer risk has been reported in patients using nonsteroidal anti-inflammatory drugs (NSAIDs), which are known to inhibit COX-2 [30, 31]. In addition, several studies have shown that different COX-2 inhibitors regulate the proliferation of many tumor cell types [32–35]. In CRC, the COX-2 specific inhibitor NS-398 reduced the proliferation of a highly invasive mouse CRC cell line (MC-26) in association with a decrease in PCNA levels [36]. Activation of stromal COX-2 signaling has also been shown to induce proliferation of human colonic epithelial cancer cells [37]. Similarly, our results demonstrate that jacalin was able to decrease the number of COX-2 positive colonic stromal cells and reduce intestinal epithelial cell proliferation in MNNG-exposed animals, suggesting that jacalin's antiproliferative effect is related to COX-2 inhibition.

COX-2 expression is usually transient and can be rapidly induced by cytokines, lipopolysaccharide (LPS), phorbol myristate acetate (PMA), or growth factors [38]. Some inflammatory factors, such as IL-1*β* and TNF-*α*, may stimulate a sustained COX-2 expression and PGE_2_ production in colonic tissues, promoting proliferation and invasiveness of colon cancer epithelial cells [39, 40]. Surprisingly, although jacalin treatment reduced COX-2 expression, high levels of TNF-*α* were detected in colon homogenates from carcinogen-exposed mice treated with the higher dose of the lectin. Given that TNF-*α* is known to induce colon cancer cell death both in vivo and in vitro [41, 42], we hypothesize that this cytokine, by inducing death of transformed cells, might be related with jacalin's chemopreventive effect. Indeed, we previously observed significant cell death rates among human colon adenocarcinoma cells (HT-29 cell line) exposed to supernatants from jacalin-stimulated macrophages, which contained high TNF-*α* levels [43].

Depending on the treatment conditions and cell types, TNF-*α* can induce both apoptotic and necrotic cell death [44]. Contrary to the well-known mechanisms involved in TNF-*α*-induced apoptosis, the signaling pathway to necrotic cell death in response to this cytokine remains elusive. Studies have shown that reactive oxygen species (ROS) may play an important role in TNF-induced necrosis [45–48]. HT-29 cells treated with NSAIDs exhibited high levels of ROS that were able to sensitize these cells to TNF-induced cell death [41, 49]. Given that the apoptosis index was not affected by jacalin treatment, we hypothesize that lectin, similar to NSAIDs, sensitizes transformed cells to TNF-*α*-induced necrosis by increasing ROS production. However, further studies are needed to unravel the precise role of TNF-*α* in jacalin's anticarcinogenic activity.

In conclusion, our results demonstrate that jacalin is able to modulate the early stages of colon carcinogenesis, inhibiting development of preneoplastic lesions. Jacalin's anticarcinogenic activity was associated with decreased colonic epithelial cell proliferation and COX-2 expression and with increased intestinal production of TNF-*α*. Our findings highlight the potential chemopreventive activity of jacalin in colorectal cancer.

## Abbreviations

ACF: Aberrant crypt foci
COX-2: Cyclooxygenase 2
CRC: Colorectal cancer
H&E: Hematoxylin and eosin
MNNG: Methyl-N′-Nitro-N-Nitroso-Guanidine
PCNA: Proliferating cell nuclear antigen
TF: Thomsen-Friedenreich antigen.
